# Separation of Hepatitis C genotype 4a into IgG-depleted and IgG-enriched fractions reveals a unique quasispecies profile

**DOI:** 10.1186/1743-422X-5-103

**Published:** 2008-09-23

**Authors:** Isabelle Moreau, Hilary O'Sullivan, Caroline Murray, John Levis, Orla Crosbie, Elizabeth Kenny-Walsh, Liam J Fanning

**Affiliations:** 1 Molecular Virology Diagnostic & Research Laboratory, Department of Medicine, Clinical Sciences Building, Cork University Hospital, Cork, Ireland; 2 Department of Gastroenterology, Cork University Hospital, Cork, Ireland

## Abstract

**Background:**

Hepatitis C virus (HCV) circulates in an infected individual as a heterogeneous mixture of closely related viruses called quasispecies. The E1/E2 region of the HCV genome is hypervariable (HVR1) and is targeted by the humoral immune system. Hepatitis C virions are found in two forms: antibody associated or antibody free.

The objective of this study was to investigate if separation of Hepatitis C virions into antibody enriched and antibody depleted fractions segregates quasispecies populations into distinctive swarms.

**Results:**

A HCV genotype 4a specimen was fractionated into IgG-depleted and IgG-enriched fractions by use of Albumin/IgG depletion spin column. Clonal analysis of these two fractions was performed and then compared to an unfractionated sample. Following sequence analysis it was evident that the antibody depleted fraction was significantly more heterogeneous than the antibody enriched fraction, revealing a unique quasispecies profile. An in-frame 3 nt insertion was observed in 26% of clones in the unfractionated population and in 64% of clones in the IgG-depleted fraction. In addition, an in-frame 3 nt indel event was observed in 10% of clones in the unfractionated population and in 9% of clones in the IgG-depleted fraction. Neither of these latter events, which are rare occurrences in genotype 4a, was identified in the IgG-enriched fraction.

**Conclusion:**

In conclusion, the homogeneity of the IgG-enriched species is postulated to represent a sequence that was strongly recognised by the humoral immune system at the time the sample was obtained. The heterogeneous nature of the IgG-depleted fraction is discussed in the context of humoral escape.

## Background

Hepatitis C is a virus affecting more than 170 million people worldwide and presents a major challenge to the health care system [1]. The virus can result in chronic hepatitis in about 50% to 80% of cases [2-4]. HCV, a member of the *Flaviviridae *family, has a linear, single stranded RNA genome of approximately 9.6 kilobases in length which encodes a polyprotein of about 3,100 amino acids [5]. Currently, seven main genotypes have been determined, which can be further divided into several distinct subtypes [5]. HCV exists within an infected individual as a dynamic population of heterogeneous but closely related variants designed as quasispecies [1,6].

The high level of diversity in HCV is primarily due to the RNA-dependent RNA polymerase, which lacks a 3'–5' proofreading function. Hence, the daughter genomes will be similar but not identical [6,7].

In a quasispecies population advantageous mutations are recurrently selected for replication where a dynamic process of continuous positive selection exists [7]. This evolution of HCV quasispecies is driven, in part, by the humoral immune system [7]. The sequence diversity exhibited during quasispecies evolution has been postulated to be related to HCV persistence and to influence HCV pathogenesis [8,7].

Although characteristically variable and postulated to be a flexible structure, the HVR1 has genetic constraints upon its amino acid composition. Penin *et al *found that while the amino acid variability of the HVR1 in response to the immune pressure is extensive, the conformation and the physicochemical properties of the HVR1 were ultimately conserved [9]. The HVR1 is primarily basic, indicative of the interactions with negatively charged molecules such as lipids, proteins or glycosaminoglycans [9,10].

Serum samples from patients infected with HCV can be fractionated by centrifugation. Studies have shown that the low density fractions, in contrast to high density fractions, are enriched for immunoglobulin (IgG) free HCV particles and plasma lipoproteins [11]. The low density fraction may represent a more highly infectious fraction, compared to the immunoglobulin G (IgG) associated fraction [12,11]. The binding of antibodies to the HCV HVR1 has been shown *in vitro *to prevent the initiation of the replication cycle in susceptible cells [13]. The bound antibody likely inhibits the engagement between the virion and the target receptor [14]; although recent evidence may suggest that antibody dependent enhancement of infection is a feature of the HCV life cycle [15]. In contrast to centrifugation, the use of a solid phase monoclonal antibody based fractionation methodology lends itself to greater selective separation of an IgG-enriched Hepatitis C virion fraction from the IgG-depleted fraction. Centrifugation based separation, with respect to IgG, can be incomplete with fraction cross contamination evident when separation is measured by RT-PCR.

As the immune system responds to the presence of HCV epitopes, the specific antibody titre rises and susceptible virions are culled from the quasispecies. This positive selection influences the emergence of escape mutants leading to the emergence of virions with altered surface glycoprotein [13]. This latter phenomenon implies that the size and heterogeneity of the fractions obtained after antibody depletion are likely to vary temporally.

## Results

### Clonal analysis and sequence data

A total of 38 clones were analysed as follows; 19 unfractionated clones, 11 IgG-depleted clones and 8 IgG-enriched clones. All the 38 sequences of 320 bp in length, encompassing the HVR1, were aligned at both the nucleotide and the amino acid level. The 19 unfractionated clones corresponded to 12 unique clones at the nucleotide level [EU482129–EU482140] and 9 species at the amino acid level. The 11 IgG-depleted clones corresponded to 8 unique clones at the nucleotide level [EU482141–EU482148] and 7 species at the amino acid level. The 8 IgG-enriched clones corresponded to 1 unique quasispecies at both the nucleotide and the amino acid level [EU482135].

**Figure 1:**
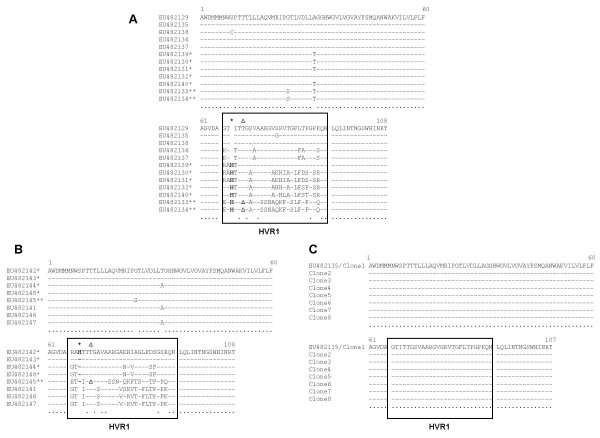
**Amino acid alignment of the individual species obtained from each fraction. **(A) unfractionated serum, (B) IgG-depleted fraction and (C) IgG-enriched fraction. The black box encloses the 27 amino acid sequence of the HVR1. The asterisk (*) indicate the position of the histidine insertion which is shown in bold (H). The (Δ) indicate the position of the deletion event when present. The closed circles indicate consensus sequence across all species.

Figure 1(A–C) shows the three different alignment profiles at the amino acid level, obtained for the unfractionated sample (A), the IgG-depleted fraction (B) and the IgG-enriched fraction (C). The quasispecies corresponding to accession number EU482135 was the subdominant quasispecies of the unfractionated sample present in 21% of clones (n = 4/19) and was recovered in 100% of the IgG-enriched clones (n = 8/8) (Figure 1C). Hence, a completely homogeneous population had been recovered for the IgG-enriched fraction. Furthermore, this quasispecies [EU482135] was not identified within the IgG-depleted fraction (Figure 1B).

26% of the unfractionated clones were identified to have an in-frame 3 nt insertion (n = 5/19), which corresponded to an inserted histidine residue at position 3 of the HVR1 [EU482130, EU482131, EU482132, EU482139, EU482140] (Figure 1A). This event also occurred in 64% of the IgG-depleted fraction (n = 7/11). These 7 IgG-depleted clones were reduced to 4 unique quasispecies at the amino acid level due to non-synonymous mutations [EU482142, EU482143, EU482144, EU482148] (Figure 1B), but were not identified within the IgG-enriched fraction (Figure 1C).

A simultaneous in-frame 3 nt indel event was also identified in 10% of the unfractionated fraction (n = 2/19) [EU482133, EU482134] (Figure 1A) and 9% of the IgG-depleted fraction (n = 1/11) [EU482145] (Figure 1B). This event correlated to an inserted histidine residue at position 3 of the HVR1 and a deleted threonine residue at position 6 of the HVR1. This event was not identified within the homogenous IgG-enriched fraction (Figure 1C).

**Figure 2:**
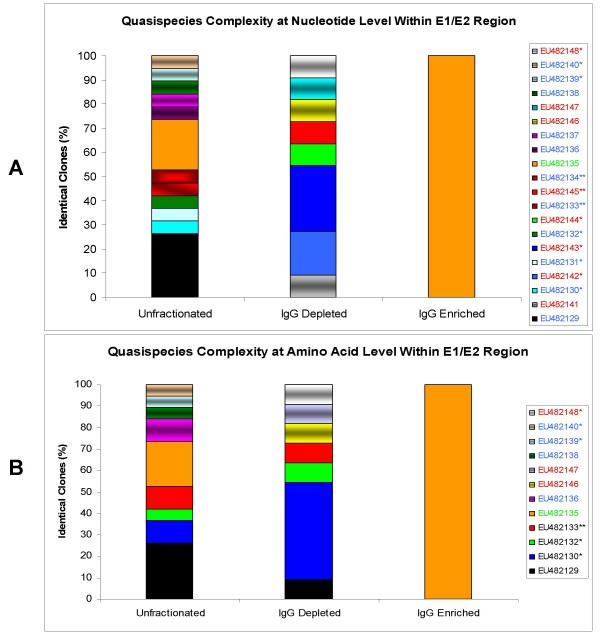
**Quasispecies complexity within E1/E2 region in the unfractionated serum, the IgG-depleted fraction and the IgG-enriched fraction:**(A) at the nucleotide level and (B) at the amino acid level. The vertical bars indicate the proportion of viral variants within each sample. Within the vertical bars, each variant is represented by a different colour. The same colour indicates identity between viral strains present in different fractions. The accession numbers and corresponding viral variant colour code of each strain are shown in the legend box where font colour corresponds to each fraction as follows: red for the unfractionated serum, blue for the IgG-depleted fraction, green for the IgG-enriched fraction [EU482135] was also the subdominant species of the unfractionated fraction), and black for quasispecies present in both unfractionated and IgG-depleted fractions. An accession number followed by an asterisk (*) indicates the presence of an insertion event within the sequence or 2 asterisks (**) indicates the presence of an indel event.

The relative frequencies of the different quasispecies within each fraction are represented in figure 2; at both the nucleotide (A) and the amino acid level (B). The completely homogeneous population of the IgG-enriched fraction is clearly demonstrated in both figure 2(A) and 2(B). At the nucleotide level there are no common species between the unfractionated fraction and the IgG-depleted fraction (Figure 2A). Non-synonymous mutations account for the subsequent overlap at the amino acid level [EU482129, EU482130, EU482132, EU482133] (Figure 2B). However, no common species between the IgG-depleted and IgG-enriched species were observed in either case.

### Phylogenetic analysis

**Figure 3:**
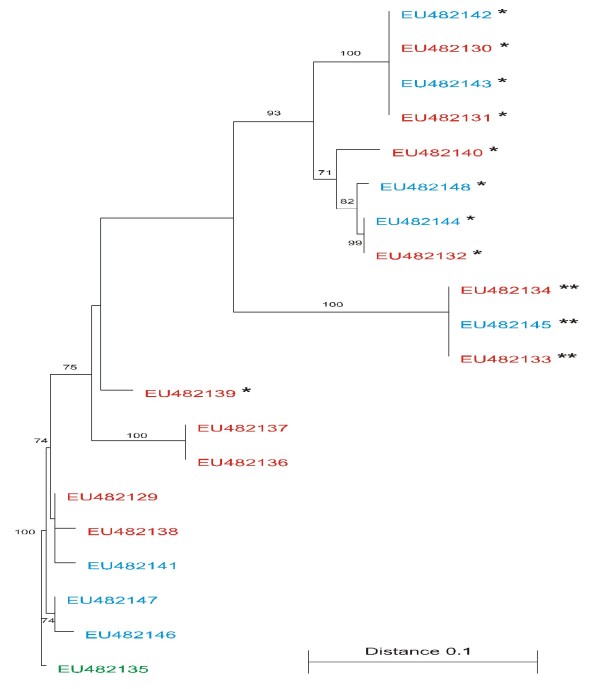
**Phylogenetic trees of all viral E1/E2 amino acid sequences encompassing the HVR1 within each fraction**. The phylogenetic tree was constructed with the Treecon software and rooted with the unique IgG-enriched species [EU482135]. The genetic distance is shown as a scale bar. A bootstrap analysis using 100 bootstrap replicates was performed to assess the reliability of each branch point. Bootstrap scores are given as percentage value. The values greater than 70% are annotated at appropriate branches. Different font colours are used to represent the different fractions: red for the unfractionated serum species, blue for the IgG-depleted species and green for the IgG-enriched species, [EU482135] was also the subdominant species of the unfractionated fraction. The species with the insertion event are represented in the figure by an asterisk (*) and the species with the indel event are represented by 2 asterisks (**).

A phylogenetic tree was constructed with the unique quasispecies of the unfractionated, the IgG-depleted and the IgG-enriched fractions at the amino acid level (figure 3). The inter-relatedness between the different quasispecies is clearly visible. The tree was rooted with the single unique quasispecies of the IgG-enriched population [EU482135], as this species was postulated to be the ancestral species from which the IgG-depleted quasispecies evolved.

## Discussion

Hepatitis C exists as a diverse population of quasispecies. The heterogeneity of the HCV genome is distributed unequally across the viral genome. Significant genomic variation occurs within the HVR1 [16]. The purpose of this investigation was to determine if IgG depletion would alter the quasispecies profile as assessed by the sequence diversity within the HVR1 region. The Qiagen Albumin/IgG Depletion Spin Column provided a quick, convenient alternative to centrifugation for the separation of HCV particles into IgG-depleted and IgG-enriched fractions.

Sequence comparison between the IgG-enriched and depleted fractions allows an insight into possible reasons why the IgG-depleted quasispecies were not associated with immunoglobulin molecules (Figure 1). A glycine residue at amino acid position 14 of the 27 amino acids of the HVR1 of the IgG-enriched quasispecies population was not evident in any of the quasispecies from the IgG-depleted fraction (Figure 1B and 1C). It is possible that a glycine at this position was important for immune recognition and that mutation enabled effective humoral immune escape. Strikingly, the replacement residue at position 14 in the depleted fraction was a polar, yet hydrophilic amino acid; lysine (K), serine (S), asparagine (N) or glutamine (Q) (Figure 1).

The observed in-frame 3 nt insertion event appears to be the first documentation of this phenomenon in Hepatitis C genotype 4a, as no sequence similarities were found when comparing these sequences against GenBank database. The significance of the inserted histidine, which was identified in the unfractionated and the IgG-depleted fraction, may lie in the ability of these diverse hypervariable regions to escape recognition by the humoral and perhaps cytotoxic arms of the immune system through disruption of dominant epitopes, although this requires further investigation.

A simultaneous in-frame 3 nt indel event was also observed in the unfractionated and IgG-depleted population at amino acid positions 3 and 6 of the HVR1 [EU482133 and EU482145], figure 1(A) and 1(B) respectively. This scenario has rarely been reported in the Hepatitis C genome [17] and, to our knowledge, never in genotype 4a isolate. The mechanism for this in-frame deletion is difficult to rationalise as there is no known ligase function associated with the RNA dependent RNA polymerase encoded by the NS5B gene of the virus. The presence of naturally occurring recombinants *in vivo*, such as 2k/1b, 2i/6p and 2k/5a, may indicate the potential for the RNA dependent RNA polymerase genome to "jump" during either positive or negative strand replication [18-20]. Intra strand switching may account to the 3 nt in-frame indel events observed here [EU482133, EU482134 and EU482145] (Figure 1A–B).

There is significant evolutionary pressure for the HVR1 region to maintain a constant length of 27 amino acids [21]. The simultaneous indel event could be accredited to this evolutionary pressure. These quasispecies [EU482133, EU482134 and EU482145] maintain a 27 amino acid length HVR1 (Figure 1A–B). However, the mutations are functionally distinct from those present in the consensus sequence of the IgG-enriched fraction. Interestingly, the deleted residue, threonine (T), is a conserved polar residue in all of the other unfractionated, IgG-enriched and IgG-depleted species [EU482129–EU482132, EU482135–EU482144 and EU482146–EU482148] (Figure 1). This specific deletion may have disrupted the consensus sequence of the epitope towards which the circulating neutralising antibodies may be directed. Castro *et al *recently reported the existence of indels in the HIV genome and suggested that a triplet repeat expansion mutational mechanism may be responsible [22]. However, the HCV HVR1 within which these indels were identified does not contain any overt repeated motifs. Mechanistically the replicases of lentiviruses and flaviviruses, which have different templates, may have unique biofunctional activities that endow the quasispecies with niche isolates which can evade immune detection that concomitantly maintain viral persistence and/or impact on pathogenicity.

It has been demonstrated that antibodies raised against the C-terminus of the HVR1 may be broadly cross-reactive and have a high capacity to capture HCV variants, indicating a conserved, partially conformational epitope [23]. The physicochemical limitations in the evolution of the HRV1 may constrain the exact nature of these insertion and indel events in the N-terminus region, thereby restricting the possibility of other dominant epitopes occurring, resulting in the persistence of a particular genetic signature within a diverse population of virions. This will require further prospective evaluation, perhaps by ultra-deep pyrosequencing [24].

Phylogenetic analysis suggests that the homogeneous IgG-enriched quasispecies [EU482135], was the ancestral species from which the variants present in the IgG-depleted fraction evolved (Figure 3). Sequence alignments indicate that the conserved residues of the IgG-depleted species within HVR1 are [----T-G-VA-------------G--QN] (Figure 1B). Interestingly, the conserved amino acid residues of the IgG-depleted fraction within HVR1 are identical to the amino acids residues of the putative ancestral species [EU482135] at the corresponding positions (Figure 1C). This is indicative of the evolutionary constraints on the sequence of the HVR1 as previously shown by Lin *et al. *[25]. Furthermore, the IgG-depleted quasispecies harbouring the indel events are the furthest evolved species from the IgG-enriched quasispecies (genetic distance of 0.180, according to the scale bar in Figure 3), whereas the quasispecies harbouring the deletion events within the IgG-depleted population represent an intermediate evolution state (genetic distance ranging from 0.153 to 0.041, according to the scale bar in Figure 3). These variants are possible progenitors of the next swarm of escape mutants.

## Conclusion

Separation of a complex mixture of antibody enriched and antibody depleted HCV particles is technically not trivial. Centrifugation based separation, with respect to IgG, can be incomplete with fraction cross contamination evident when separation is measured by RT-PCR. The use of a solid phase monoclonal IgG depletion strategy provides a fast and relatively simple method for separation of HCV viral particles from a serum sample. We have demonstrated that an IgG-depleted fraction can be molecularly more diverse than the quasispecies profile of the IgG-enriched fraction. The IgG-depleted fraction was populated with genomes with an insertion and indel events. This is the first documentation of such occurrences in Hepatitis C genotype 4a. These quasispecies are likely to represent humoral escape mutants and suggest that separations based on viral-antibody complexes will likely exhibit temporal patterns of change.

## Methods

### Serum sample

A serum sample from a panel of viraemic sera positive for HCV genotype 4a was randomly selected. The VERSANT^® ^HCV Genotype Assay (LiPA) was used to confirm the genotype of this HCV sample [26]. The viral load measurement was previously determined by use of Ampliprep/COBAS-TaqMan 48 platform (Roche Diagnostics, UK) and was found to be 6.37 log_10 _IU/ml. A waiver of consent was provided by Clinical Research Ethics Committee of the Cork Teaching Hospitals as the sample used in this study was surplus to requirements following diagnostic investigations.

### Serum sample fractionation into IgG-depleted and IgG-enriched fractions

The original serum sample was separated into IgG-enriched and IgG-depleted fractions using Albumin/IgG Depletion Spin Columns following the Qproteome Albumin/IgG Depletion protocol (Catalogue No: 37521, QIAGEN, UK). This column exploits the use of monoclonal antibodies which can bind human serum albumin and human IgG with high affinity and specificity.

As per manufacturers protocol, 25 μl of serum was diluted in 75 μl of dilution buffer (Phosphate Buffered Saline, PBS). The diluted serum sample was applied to the column. The flow-through was collected through centrifugation and contained the IgG-depleted fraction. The column was then washed twice with PBS, collecting eluate by centrifugation. These eluates were combined in order to increase the amount of IgG-depleted viral particles recovered. Two additional wash steps were performed with PBS in order to limit contamination of the IgG-enriched fraction with any IgG-free viral particles. These eluted volumes were discarded.

250 μl of Lysis/Binding Buffer of the MagNA Pure LC Total Nucleic Acid Isolation Kit (Roche Diagnostics, UK) was then added directly to the column to lyse the IgG-enriched viral particles. The column was then inverted and mixed on the end-over-end shaker (DYNAL sample mixer) for 5 minutes at room temperature followed by centrifugation. Another 250 μl of Lysis/Binding Buffer (Roche Diagnostics, UK) was then added and the mixing step and centrifugation was repeated. These two fractions were then combined.

### HCV RNA extraction

HCV RNA was extracted on the MagNA Pure LC (Roche Diagnostics Ltd. UK,) according to the MagNA Pure LC Total Nucleic Acid Isolation Kit protocol (Catalogue No: 03038505001, Roche Diagnostics Ltd., UK) from 25 μl of an unfractionated serum sample, in addition to the IgG-depleted eluate and the lysed material of the IgG-enriched fraction.

### Amplification of E1/E2 region encompassing the HVR1

Reverse Transcription was performed as previously described by [27].

Amplification of the E1/E2 region encompassing the HVR1 was performed using nested primers and hence, Set I previously described by Lin *et al*, resulting in a 320 bp fragment extending from nucleotides 1234–1553 according to isolate ED43 reference strain genotype 4a (GenBank accession no Y11604). The primer sequences used were as follows (5' to 3'): outer forward, OF (I), ATGGCATGGGATATGAT; outer reverse, OR (I), AAGGCCGTCCTGTTGA; inner forward, IF (I), GCATGGGATATGATGATGAA; inner reverse, IR (I), GTCCTGTTGATGTGCCA. The PCR reactions were performed with the proofreading *Pwo *DNA polymerase (Roche Molecular Biochemicals, UK) as previously described by [27]. All E1/E2 amplicons were gel purified and cloned into Zero Blunt^® ^TOPO^® ^PCR Cloning Kit (Catalogue No: K2895-40, Invitrogen, Belgium). Plasmid DNA purification was performed using the QIAprep Miniprep Kit (Catalogue No: 27104, Qiagen, UK) before sequencing.

### Sequence analysis of the E1/E2 region

Positive clones were sequenced by MWG-Biotech  Germany. E1/E2 320 bp sequences were aligned using ClustalW2 , MultAlin  and analysed using the NCBI BLAST N (nucleotide) web program .

### Phylogenetic analysis

A phlyogenetic tree was constructed by use of TREECON software . The cut-off bootstrap value was 70% with 100 replicates.

### Accession numbers

The sequences reported in this study have been assigned the following GenBank accession numbers: unfractionated [EU482129–EU482140] and IgG-depleted [EU482141–EU482148]. The single unique species identified for the IgG-enriched fraction had been identified within the unfractionated population [EU482135].

## Competing interests

The authors declare that they have no competing interests.

## Authors' contributions

IM supervised the experiments and contributed to data analysis and preparation of manuscript. HO'S performed all the experiments and contributed to data analysis and preparation of manuscript. CM contributed to the experiments and data analysis. JL determined qualitative, quantitative and genotype of clinical specimens described here. OC and EKW are clinicians who manage HCV at Cork University Hospital. LJF supervised the project and assisted with preparation of manuscript. All authors have read and approved the present manuscript.
